# Genome-scale CRISPR/Cas9 screening reveals the role of *PSMD4* in colibactin-mediated cell cycle arrest

**DOI:** 10.1128/msphere.00692-24

**Published:** 2025-02-07

**Authors:** Michael W. Dougherty, Ryan M. Hoffmann, Maria C. Hernandez, Yougant Airan, Raad Z. Gharaibeh, Seth B. Herzon, Ye Yang, Christian Jobin

**Affiliations:** 1 Department of Medicine, University of Florida College of Medicine, Gainesville, Florida, USA; 2 Department of Chemistry, Yale University, New Haven, Connecticut, USA; 3 Department of Molecular Genetics and Microbiology, University of Florida, Gainesville, Florida, USA; 4 Departments of Pharmacology, Yale University, New Haven, Connecticut, USA; 5 Department of Infectious Diseases and Immunology, University of Florida College of Medicine, Gainesville, Florida, USA; 6 Department of Anatomy and Cell Biology, University of Florida College of Medicine, Gainesville, Florida, USA; University of Michigan-Ann Arbor, Ann Arbor, Michigan, USA

**Keywords:** colibactin, *Escherichia coli*, *Klebsiella pneumoniae*, *PSMD4*, cell cycle arrest, colorectal cancer

## Abstract

Colibactin is a genotoxic secondary metabolite produced by certain *Enterobacteriaceae* strains that populate the intestine and produces a specific mutational signature in human colonocytes. However, the host pathways involved in colibactin response remain unclear. To address this gap, we performed genome-wide CRISPR/Cas9 knockout screens and RNA sequencing utilizing live *pks^+^* bacteria and a synthetic colibactin analog. We identified 20 enriched genes with a MAGeCK score of >2.0 in both screens, including proteasomal subunits (e.g., *PSMG4* and *PSMD4*), RNA processing factors (e.g., *SF1* and *PRPF8*), and RNA polymerase III (e.g., *CRCP*), and validated the role of *PSMD4* in colibactin sensitization. *PSMD4* knockout in HEK293T and HT-29 cells promoted cell viability and ameliorated G2-M cell cycle arrest but did not affect the amount of phosphorylated H2AX foci after exposure to synthetic colibactin 742. Consistent with these observations, *PSMD4*^−/−^ cells had a significantly higher colony formation rate and bigger colony size than control cells after 742 exposure. These findings suggest that *PSMD4* regulates cell cycle arrest following colibactin-induced DNA damage and that cells with *PSMD4* deficiency may continue to replicate despite DNA damage, potentially increasing the risk of malignant transformation.

**IMPORTANCE:**

Colibactin has been implicated as a causative agent of colorectal cancer. However, colibactin-producing bacteria are also present in many healthy individuals, leading to the hypothesis that some aspects of colibactin regulation or host response dictate the molecule’s carcinogenic potential. Elucidating the host-response pathways involved in dictating cell fate after colibactin intoxication has been difficult, partially due to an inability to isolate the molecule. This study provides the first high-throughput CRISPR/Cas9 screening to identify genes conferring colibactin sensitivity. Here, we utilize both bacterial infection and a synthetic colibactin analog to identify genes directly involved in colibactin response. These findings provide insight into how differences in gene expression may render certain individuals more vulnerable to colibactin-initiated tumor formation after DNA damage.

## INTRODUCTION

Colibactin is a genotoxin produced by bacteria carrying the *pks* gene island and implicated in colorectal cancer (CRC) development via its mutagenic activity in colonic epithelial cells (1–5). Despite colibactin’s clinical significance, the genotoxin’s mode of action as a potent DNA interstrand cross-linking (ICL) agent has only recently been defined through a combination of chemical and biological studies (6, 7). Consistent with this ICL activity, *pks^+^ Escherichia coli* infection promotes the recruitment of Fanconi anemia (FA) proteins to phosphorylated H2AX (γH2AX) sites (7), the formation of double-strand breaks (1), the activation of non-homologous end-joining DNA repair pathway (8), and the induction of autophagy that promotes tumor formation (9). Despite these observations, no clear host-response pathways have been identified as essential to colibactin sensitization, limiting our ability to predict host genetic factors that may increase the risk of CRC resulting from the presence of *pks^+^* bacteria in the intestinal tract. This is a significant gap in knowledge that may partially explain why approximately 20% of healthy people can be colonized by *pks^+^ E. coli* (2) without tumor formation, or the presence of the *pks* gene island in the common probiotic *E. coli* strain Nissile 1917 (10) without documented carcinogenic consequences. To more accurately assess the risks associated with *pks^+^* bacterial infection, a better understanding of specific colibactin-associated risk factors is essential.

Mechanistic insights have been partially limited by an inability to isolate colibactin from *pks^+^* microbes. Colibactin is produced in vanishingly small quantities, and evidence suggests it is chemically unstable (11). We recently described the synthesis of a stable colibactin analog, colibactin 742, that addresses these challenges (12) and validated the molecule’s DNA-damaging and mutagenic activities in human cell lines and colonic organoids (13). However, high-throughput analyses comparing the cellular responses to this synthetic molecule and live bacterial infection do not exist. Here, we utilized this synthetic colibactin mimetic and a live strain of *pks^+^ Klebsiella pneumoniae* to identify essential colibactin-response pathways using global transcriptomics and genome-wide CRISPR/Cas9 screening. We show that there is a core colibactin response recapitulated by colibactin 742 in normal human colonic cells and use a genome-wide CRISPR/Cas9 screen to show that genes involved in transcription and proteasomal regulation of cellular function sensitize cells to the cytotoxic effects of colibactin. We validated a candidate identified from our CRISPR/Cas9 screen, *PSMD4*, demonstrating that this gene is essential for the induction of G2-M cell cycle arrest but not DNA damage after colibactin 742 exposure.

## RESULTS

**Fig 1:**
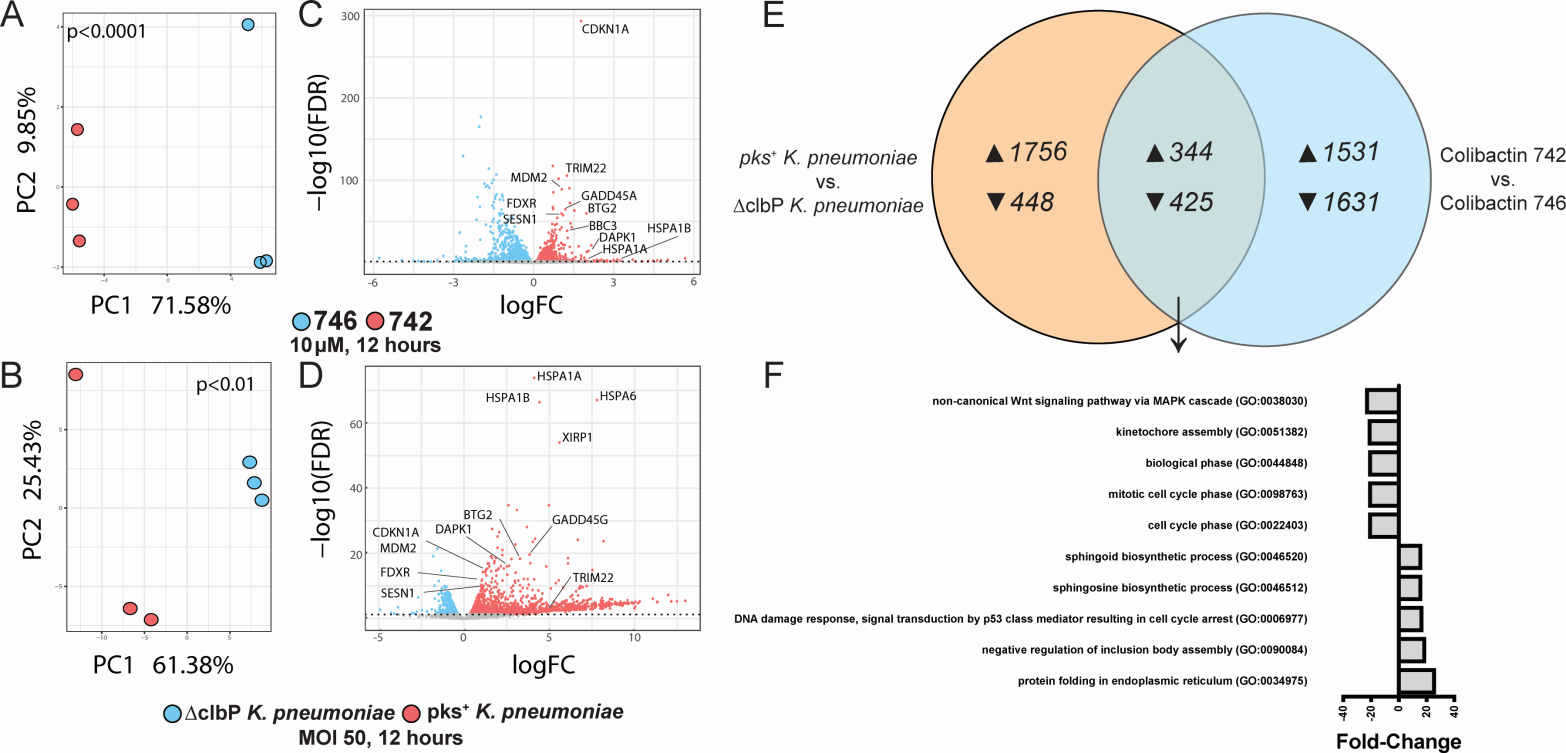
Colibactin 742 and *pks*^+^ *Klebsiella pneumoniae* activate transcriptional pathways associated with cell cycle arrest, DNA damage response, and ER stress. (**A and B**) Principal component analysis of global transcriptomic data in FHC cells treated with colibactin 742 or the inactive colibactin 746 (**A**) or with *pks^+^ K. pneumoniae* or an isogenic ΔclbP *K. pneumoniae* mutant (**B**). (C and D) Volcano plot of differential gene expressions in FHC cells treated with colibactin 742 or the inactive colibactin 746 (**C**) and with *pks^+^ K. pneumoniae* or an isogenic ΔclbP *K. pneumoniae* mutant (**D**); genes with an FDR of <0.05 enrichment in each treatment group denoted by color. (**E**) Venn diagram of significant (FDR < 0.05) differentially expressed genes in each comparison (742 vs 746, *pks^+^ K. pneumoniae* vs ΔclbP *K. pneumoniae*). (**F**) PANTHER overrepresentation analysis performed on overlapping differentially expressed genes. FC, fold change.

We previously showed that colibactin 742 causes DNA damage and mutagenesis in colonic epithelial cells (13). However, the genetic pathways implicated in colibactin host response remain poorly defined. We first sought to identify similarities between the cellular responses to the synthetic genotoxin and colibactin-producing bacteria. For these studies, we used a clinical neonatal *Klebsiella pneumoniae* isolate (51–5) that produces colibactin and promotes tumor formation in murine models (14). We confirmed by PCR that *K. pneumoniae* lacks the gene *cnf1* encoding cytotoxic necrotizing factor-1 that has been shown to modulate the carcinogenic effects of colibactin (15) (Fig. S1). We assessed changes in gene expression after infection or exposure to colibactin or *K. pneumoniae* 51–5 relative to cells treated with an inactive form of colibactin (colibactin 746) or infected by an isogenic *K. pneumoniae* mutant that does not produce colibactin (Δ*clbP K. pneumoniae*). Transcriptomic analysis showed that treatment with colibactin 742 and *pks^+^ K. pneumoniae* 51–5 significantly altered global transcriptional profiles in the human colon cell line FHC, increasing the expression of core colibactin-response genes associated with DNA damage response (e.g., *MDM2*, *FDXR*, and *BBC3*), endoplasmic reticulum (ER) stress (e.g., *SESN1*), and cell cycle arrest (e.g., *BTG2* and *CDKN1A*), consistent with our previous study (13) (Fig. 1A through D). To better characterize genes specifically associated with colibactin intoxication, we cross-referenced differentially expressed genes (false discovery rate [FDR] < 0.05) after colibactin 742 and *pks^+^ K. pneumoniae* exposure and identified 344 and 425 shared genes upregulated and downregulated, respectively (Fig. 1E). Overlapping gene signatures were attributed to the upregulation of DNA damage response and protein folding in the ER gene ontology (GO) pathways (Fig. 1F). Consistent with colibactin’s ability to cause rapid cell cycle arrest, downregulated overlapping gene signatures were associated with a downregulation of WNT signaling and the mitotic cell cycle (Fig. 1F). These data suggest that synthetic colibactin and *pks^+^ K. pneumoniae* activate a core cellular response consistent with colibactin’s DNA-damaging activity.

**Fig 2:**
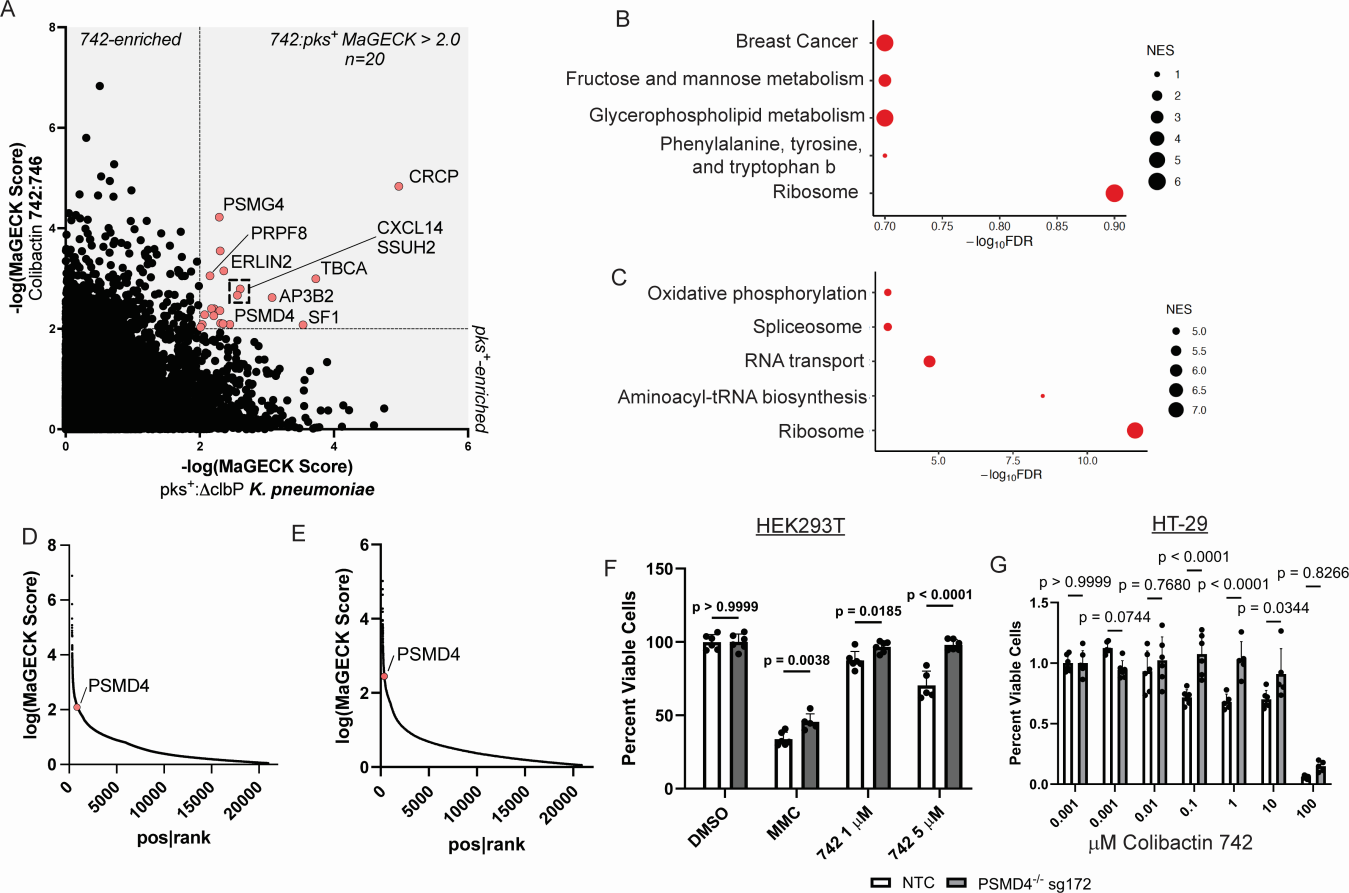
The 26S proteasome subunit ubiquitin receptor, *PSMD4*, facilitates colibactin sensitivity. (**A**) Positive enrichment scores (MaGECK robust rank aggregation [RRA]) from genome-wide CRISPR/Cas9 screens comparing colibactin 742 vs colibactin 746 (*y*-axis) and *pks^+^ K. pneumoniae* vs ΔclbP *K. pneumoniae* (*x*-axis), with colored points representing genes with MaGECK scores of >2.0 in both screens. (**B and C**) MaGECK RRA KEGG pathway analysis of positively enriched genes in *K. pneumoniae* (**B**) and synthetic colibactin (**C**) CRISPR/Cas9 screens. (**D and E**) MaGECK RRA-positive enrichment ranking from CRISPR/Cas9 screens utilizing synthetic colibactins (**D**) and *K. pneumoniae* (**E**) showing the position of *PSMD4* in red. (**F**) Cell viability after 48 h treatment with dimethyl sulfoxide (DMSO), mitomycin C, or colibactin 742 at the indicated concentration, in non-target control (NTC) or *PSMD4*^−/−^ HEK293T cell populations. *n* = 6 from a single experiment, two-way analysis of variance (ANOVA) with Tukey’s post hoc test. (**G**) Cell viability after 48 h treatment with colibactin 742 at the indicated concentration in non-target control or *PSMD4*^−/−^ HT-29 cell populations. *n* = 4–6 from a single experiment, two-way ANOVA with Tukey’s post hoc test.

Next, we sought to identify genes that confer colibactin sensitivity by performing genome-wide CRISPR/Cas9 knockout screens using the GeCKO v2 sgRNA library in HEK293T cells. We performed two positive CRISPR screens using an approximate LC50 dose of colibactin 742 (5 μM, continuous dose) or *pks^+^ K. pneumoniae* infection (multiplicity of infection [MOI] 50, 4 h infection cycles), quantifying positively enriched genes in the resultant populations (after three passages, 12–14 days) relative to a control population treated with an inactive form of colibactin (colibactin 746) or infected by Δ*clbP K. pneumoniae*. To identify candidates for single-target validation, we identified 20 genes with a MAGeCK score of >2.0 in both screens (Fig. 2A), including genes associated with RNA processing (e.g., *SF1*, *PRPF8*, and *CRCP*), the proteasome (e.g., *PSMG4* and *PSMD4*), and ER stress (e.g., *ERLIN1*). Both screens’ most significantly enriched pathways were associated with RNA processing (Fig. 2B and C), suggesting that colibactin sensitization was related to transcription or translation, consistent with the transcriptional strand bias observed in the colibactin-associated mutational signature (4). We performed single-target validation on the calcitonin gene-related peptide receptor (*CRCP*), that also functions as a component of RNA polymerase III and is the most enriched guide in both screens (Fig. 2A). However, after confirming knockout in HEK293T cells by Western blot (Fig. S2A), we observed no significant difference in colibactin resistance (Fig. S2B) or G2-M cell cycle arrest (Fig. S2C). The lack of effect from *CRCP* knockout alone on these cell responses suggests that the enrichment of *CRCP* knockout cells observed in our screen is likely a result of interactions between *CRCP* knockout cells and cells with other genetic mutations; e.g., the latter population produces certain unidentified factors that confer competitive growth benefit to the former.

To determine our next lead candidate, we cross-referenced our 20 genes with a prior study using genome-wide CRISPR screens to identify genes associated with 27 genotoxins (16). We found that one target from our screen, the proteasome 26S subunit ubiquitin receptor (*PSMD4*), was highly enriched in both treatment conditions (Fig. 2A, D, and E), as well as in screens utilizing duocarmycin and bleomycin (16), two DNA alkylating agents that bind the minor groove of DNA similar to colibactin (5, 17, 18). We generated single-target *PSMD4* knockout populations in HEK293T cells using two guide RNAs from the GeCKOv2 library. Moreover, we quantified the proportion of knockouts using tracking of indels by decomposition (TIDE) amplicon sequencing (19), resulting in two knockout populations with total knockout efficiencies of 67.0% (*PSMD4* sg171) and 72.1% (PSMD sg172) (Fig. S3A). We next generated *PSMD4* knockouts in colonic epithelial cells (HT-29) and confirmed knockout efficacy using TIDE as previously described, yielding two knockout populations with total knockout efficiencies of 65.4% (*PSMD4* sg171) and 77.8% (*PSMD4* sg172) (Fig. S3B). To confirm the results of our genome-wide screen, we measured changes in cell viability after 48 h of treatment with colibactin 742. We found that both *PSMD4* knockout HEK293T cell populations exhibited enhanced survival after 5 µM colibactin 742 treatment (Fig. 2F; Fig. S4A). To validate this finding in human colonic epithelial cells, we measured changes in cell viability in *PSMD4*^−/−^ sg172 HT-29 cells after 48 h of treatment with colibactin 742. We found that in HT-29 cells, *PSMD4* knockout populations exhibit enhanced survival after 0.1, 1.0, and 10.0 µM colibactin 742 treatment (Fig. 2G).

**Fig 3:**
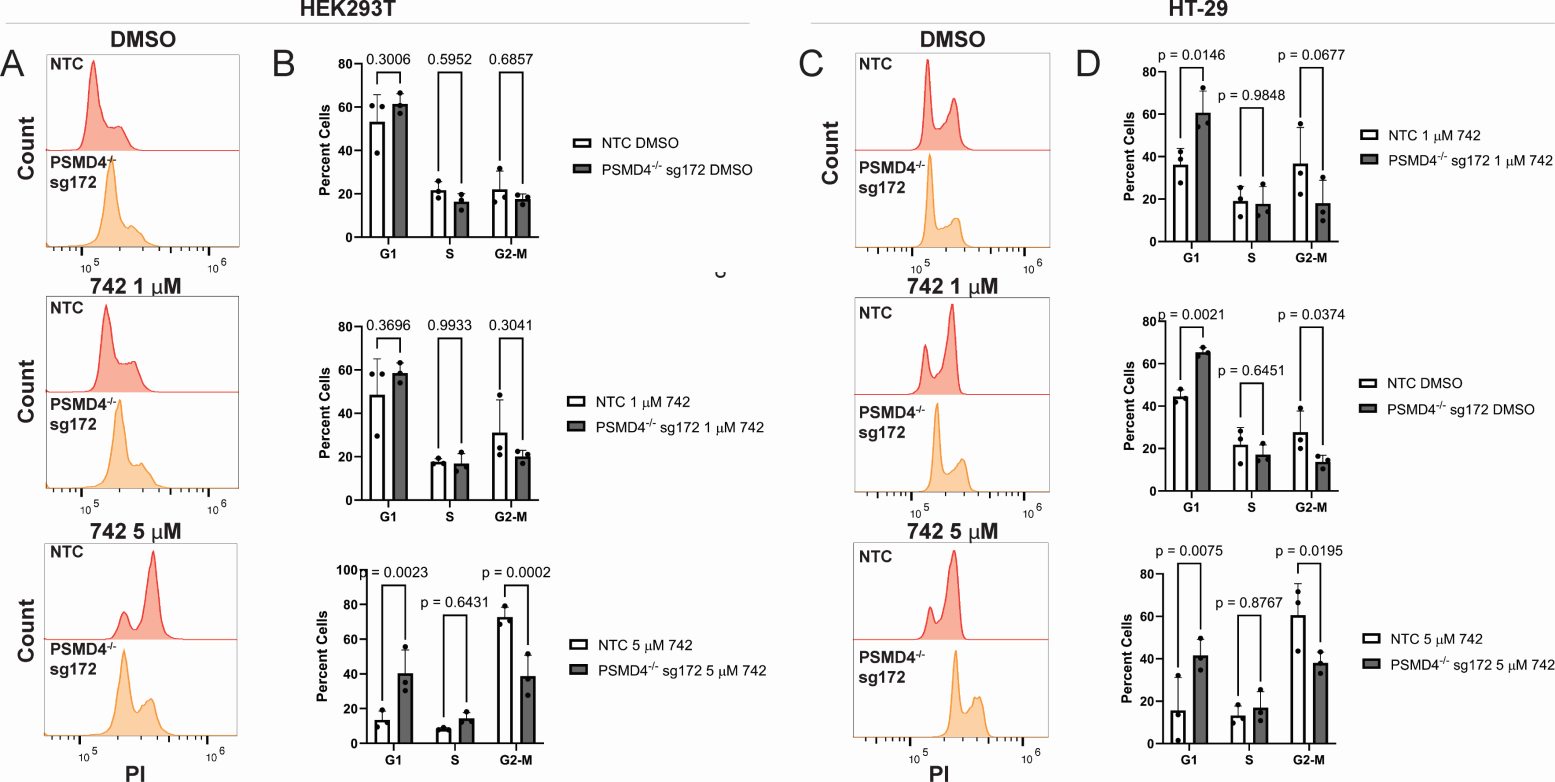
*PSMD4* facilitates colibactin-induced G2-M cell cycle arrest. (**A**) Flow cytometry histograms of propidium iodide (PI) staining in non-target control (NTC) or *PSMD4*^−/−^ HEK293T cells after 24 h DMSO, or colibactin 742 treatment. Data are representative of three independent experiments. (**B**) Percentage of NTC or *PSMD4*^−/−^ HEK293T cells in G1, S, and G2-M phase after treatment with 24 h DMSO or colibactin 742 as shown in panel** A**. *n* = 3 independent experiments, two-way ANOVA with Tukey’s post hoc test. (**C**) Flow cytometry histograms of propidium iodide staining in NTC or *PSMD4*^−/−^ HT-29 cells after 24 h DMSO or colibactin 742 treatment. Data are representative of three independent experiments. (**D**) percentage of NTC or *PSMD4*^−/−^ HT-29 cells in G1, S, and G2-M phase after treatment with DMSO or colibactin 742 as shown in panel **A**. *n* = 3 independent experiments, two-way ANOVA with Tukey’s post hoc test.

**Fig 4:**
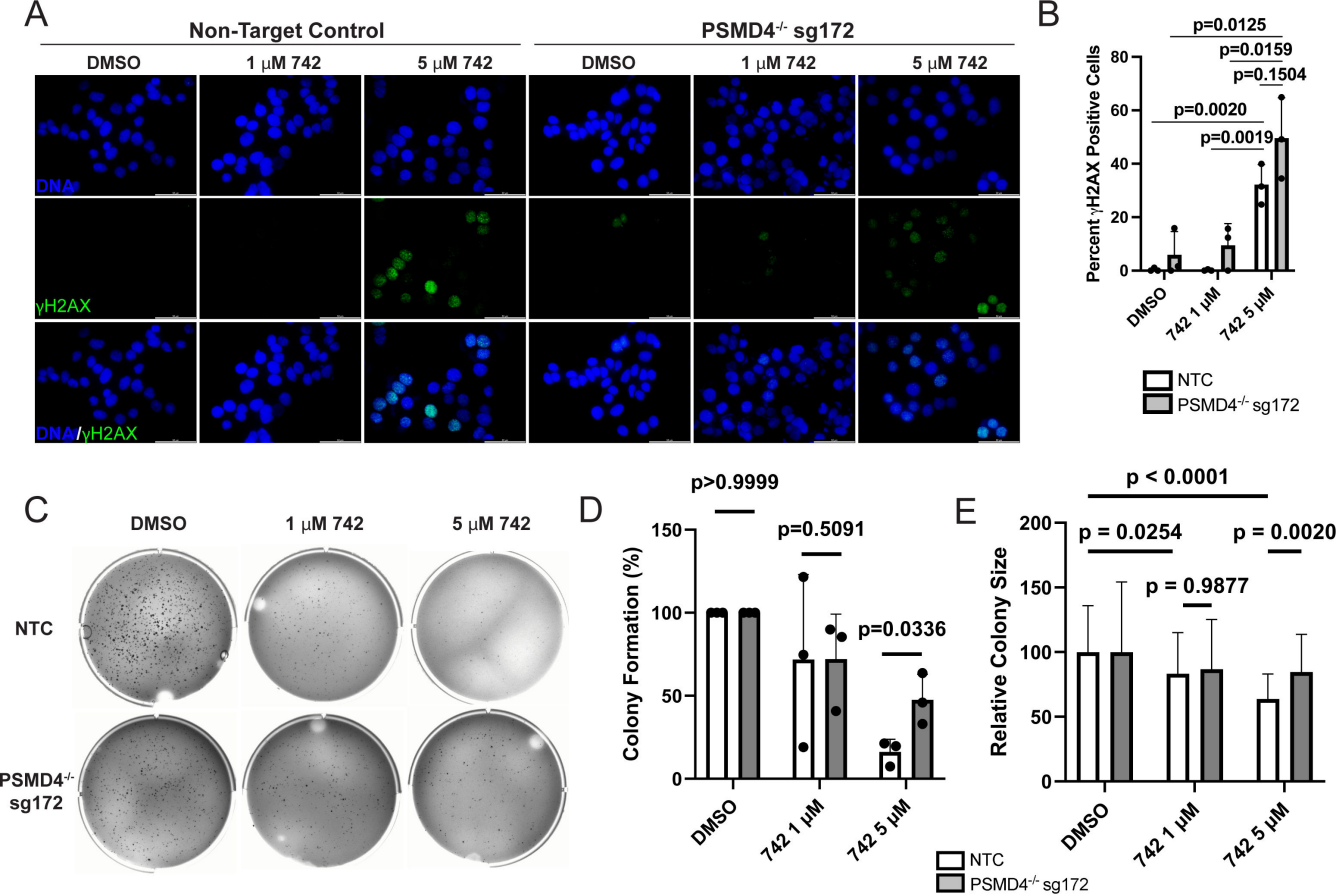
Colibactin inhibits colony formation, but not DNA damage, in a *PSMD4*-dependent manner. (**A**) Representative images of non-target control (NTC) and *PSMD4*^−/−^ HT-29 cells after 24 h DMSO or colibactin 742 treatment before visualizing DNA strand breaks by immunofluorescent staining using γH2AX antibodies. Data are representative of three independent experiments. Scale bar, 100 µm. (B) The percentage of cells with >5 γH2AX foci as shown in panel **A** was quantified. *n* = 3 independent experiments, with each datapoint representing the average of three to six ×40 fields of view from a single experiment; two-way ANOVA with Tukey’s post hoc test. (**C**) Representative images of colony formation in NTC and *PSMD4*^−/−^ HT-29 cells soft agar 21 days after 24 h DMSO or colibactin 742 treatment. (**D**) Quantification of the relative number of colonies as shown in panel **C**, with each data point representing the number of colonies in a single 9.6 cm^2^ well normalized to the number of colonies formed after DMSO treatment. *n* = 3 independent experiments, unpaired *t*-test. (**E**) Quantification of the relative diameter of colonies as shown in panel** C**, *n* = 90 randomly selected colonies pooled from three independent experiments (30 colonies per experiment), two-way ANOVA with Tukey’s post hoc test.

G2-M cell cycle arrest is a well-characterized response to colibactin intoxication (2, 3) and an important step in preserving genomic integrity as cells pause to repair damaged DNA (20). We hypothesized that the increased proportion of viable cells observed in *PSMD4* knockout populations may be due to an inadequate induction of cell cycle arrest after colibactin intoxication. Consistent with this hypothesis, we found that *PSMD4* knockout significantly reduced the proportion of cells in G2-M arrest after 5 µM colibactin 742 treatment in HEK293T cells from 72.77% (non-target control [NTC]) to 38.73% (*PSMD4*^−/−^ sg172, *P* = 0.0002) (Fig. 3A and B). We found that *PSMD4* knockout significantly increased the percentage of G1 cells in dimethyl sulfoxide (DMSO)-treated cells (NTC, 44.50%; *PSMD4*^−/−^ sg172, 65.43%, *P* = 0.0021; Fig. 3C and D), and the percentage of G2-M arrested cells after 5 µM colibactin 742 treatment (NTC, 60.53%; *PSMD4*^−/−^ sg172, 38.03%, *P* = 0.0195; Fig. 3C and D). Using a second guide targeting *PSMD4* (sg171), we observed similar resistance to colibactin 742-induced G2-M cell cycle arrest in HEK293T (Fig. S4B) and HT-29 (Fig. S4C) cells. Despite significantly reduced G2-M cell cycle arrest in *PSMD4*^−/−^ cells, we found no significant difference in the proportion of senescent cells when comparing NTC and *PSMD4*^−/−^ cells after colibactin treatment (Fig. S4D and E). To determine if this phenotype was due to a reduction in colibactin-induced DNA damage, we quantified the amount of DNA damage after colibactin exposure by measuring the number of γH2AX-positive cells after colibactin 742 treatment. We found that while 5 µM colibactin 742 treatment significantly increased the proportion of γH2AX-positive cells in both NTC and *PSMD4*^−/−^ sg172 cells compared to DMSO treatment, there was no difference in the level of DNA damage between NTC and *PSMD4*^−/−^ sg172 cells after 1 or 5 µM colibactin 742 treatment (Fig. 4A and B).

To determine if *PSMD4* knockout functionally alters the malignant potential of human colonic cells after colibactin exposure, we quantified the relative clonogenic colony-forming potential of NTC and *PSMD4*^−/−^ sg172 cells in soft agar after colibactin intoxication (Fig. 4C). We found that DMSO-treated *PSMD4* knockout populations formed fewer and smaller colonies than NTC cells (Fig. S5A and B). However, a significantly higher proportion of *PSMD4*^−/−^ sg172 cells retained colony-forming capacity than NTC cells after treatment with 5 µM colibactin 742 ((NTC, 16.50%; *PSMD4*^−/−^ sg172, 47.57%, *P* = 0.0336; Fig. 4D). Similarly, colibactin 742 treatment significantly reduced relative colony size in NTC cells but not *PSMD4* knockout populations, and *PSMD4* knockout populations formed relatively larger colonies than NTC cells after 5 µM colibactin 742 treatment (Fig. 4E). To determine if this observation was due to alterations in proliferation after colibactin exposure in *PSMD4*^−/−^ cells, we compared BrdU incorporation in NTC and *PSMD4*^−/−^ cells after 1 and 5 µM colibactin 742. We found that 5 µM colibactin 742 showed a trend toward reduced proliferation in NTC cells (*P* = 0.0661) but not *PSMD4*^−/−^ cells (*P* = 0.9462), and that the proportion of BrdU cells was significantly higher in *PSMD4*^−/−^ cells after 1 µM (*P* < 0.0001) and 5 µM (*P* = 0.0078) colibactin treatment (Fig. S5C). Overall, these data suggest that *PSMD4* is required to inhibit cell cycle arrest after colibactin exposure but does not impact colibactin-induced DNA damage.

## DISCUSSION

Epidemiological associations between bacteria carrying the *pks* gene island and CRC patients (2, 21–23), as well as the identification of a specific mutational signature derived from *pks^+^ E. coli* infection in a subset of CRC patients (4), have led to the hypothesis that these bacteria may play a causative role in tumorigenesis. While a growing body of literature investigates how other bacteria (24, 25) or environmental conditions in the gut (26–31) may influence the genotoxic and carcinogenic capability of colibactin-producing bacteria, host genetic pathways impacting cellular responses to the genotoxin remain less well characterized (32). Our results demonstrate that infection with *pks^+^* bacteria or a synthetic colibactin mimetic activates a core transcriptional response, namely, the upregulation of ER stress, p53-mediated DNA damage response pathways, and the downregulation of pathways facilitating the mitotic cell cycle. These findings are consistent with our previous transcriptional analyses of colibactin 742 (13), suggesting that this compound accurately recapitulates the effects of *pks^+^* bacterial infection. Furthermore, our observation that cells with mutations in genes associated with ribosome function were depleted in our CRISPR screen suggests that colibactin’s cytotoxic effects may be more pronounced in cells with defective gene expression or replication. Indeed, several recent studies have highlighted that cellular responses to DNA damage vary with cell cycle stage (33, 34). Similarly, another bacterial genotoxin, the cytolethal distending toxin, has been shown to cause replicative stress that preferentially induces DNA damage responses during the mitotic S phase (35, 36). Thus, it is possible that the enriched genes observed in our CRISPR/Cas9 screen are not colibactin specific but instead that their deletion reduces the rate of DNA replication or transcription, thus dampening the recognition of colibactin-DNA lesions and the induction of G2-M cell cycle arrest. This hypothesis is supported by our observation that in human colonic cancer cells, *PSMD4* deletion results in a significantly higher proportion of cells in the G1 phase under control conditions (DMSO). Furthermore, while *PSMD4* deletion prevents a relative decrease in colony formation after colibactin treatment, we observed a significant decrease in the absolute colony-forming capacity of these cells in DMSO-treated groups. These findings are consistent with the observation that the colibactin single-base substitution signature exhibits a strong transcriptional strand bias (4), suggesting that transcription-coupled repair may be a common mechanism by which cells recognize colibactin lesions and activate cell cycle arrest or apoptotic pathways if the DNA damage is beyond repair.

Alternatively, DNA-damaged cells that continue through the cell cycle may have a higher likelihood to carry DNA mutations as avoiding G2-M cell cycle arrest may not afford adequate time to repair the damaged DNA (37). We have previously shown that colibactin 742 intoxication causes high levels of cell cycle arrest, restricts clonogenic growth, and that repeated exposure increases mutational burden but restricts the growth of these cells in murine xenografts (13). Importantly, we observed similar levels of DNA damage in cells lacking *PSMD4* but a higher relative capacity for clonogenic growth than NTC cells. These findings suggest *PSMD4*^−/−^ cells may accumulate more mutations, and future studies will investigate the mutational load in cells deficient and proficient in *PSMD4*. Our findings partially explain the factors that predispose specific colonic epithelial cells to become cancerous after *pks^+^* bacterial infections. Moreover, this work suggests that patients with dysregulated *PSMD4* expression or other genes identified in our CRISPR/Cas9 screen that play essential roles in transcription and translation may be more susceptible to CRC, resulting from *pks^+^* bacterial infections.

Our findings suggest that differences in PSMD4 expression modulate epithelial cell responses to colibactin. While *PSMD4-*expressing cells are susceptible to colibactin-induced cell cycle arrest, cells lacking *PSMD4* bypass the checkpoint while still acquiring DNA damage. Because our studies compared *PSMD4* knockouts to wild-type expressing cells, more studies are needed to determine if similar differences in colibactin response in epithelial cells are present when *PSMD4* is expressed at a higher or lower level. While complete loss of *PSMD4 in vivo* is unlikely, differences in *PSMD4* expression in epithelial cells are variable. For example, high proteasome activity or ubiquitin ligase mutations that interrupt cell cycle inhibiting processes can promote malignancy in colonic cancer cells (38, 39). Thus, our findings suggest that differences in *PSMD4* expression, or proteasome-ubiquitin processes more broadly, may lead to different cell fates after colibactin exposure. Future research is needed to determine how *PSMD4* expression varies within colonic epithelial cells or cancer cells, and whether these differences impact cellular responses to colibactin or its carcinogenic potential *in vivo*. These findings have several important implications concerning colibactin’s carcinogenic risk, patient stratification, and the molecule’s potential clinical applications. First, identifying host-response factors that sensitize cells to apoptosis or senescence after colibactin exposure may allow clinicians to identify patients at higher risk of malignant transformation after *pks^+^* bacterial infections using clinical gene expression profiling. However, this would require screening for and identifying individuals colonized by *pks^+^* bacteria or those in which the colibactin mutational signature is detected by whole-genome sequencing as evidence of a historical infection. While these data shed light on host factors that may increase the likelihood of developing CRC after colibactin exposure, future studies are needed to determine the mechanism by which *PSMD4* sensitizes cells to colibactin and if this response is specific to the toxin or a more ubiquitous response to DNA alkylating agents or genotoxins. Our finding that PSMD4 deletion attenuates G2-M cell cycle arrest and the anti-proliferative effects of colibactin but does not alter cellular senescence suggests that PSMD4 may play a critical role in cellular pathways that restrict proliferation after DNA damage from colibactin cross-links. Several studies have demonstrated that the ubiquitin-proteasome system and *PSMD4* specifically are essential for the activation of nucleotide excision repair (40) or activation of the FA pathway used to resolve DNA cross-links (41). Collectively, these findings suggest *PSMD4* may play a direct role in the activation of downstream events triggered by colibactin-induced DNA damage. In conclusion, our findings identify a set of colibactin-sensitizing gene candidates in human cells that may be leveraged to gain further insight into the cellular factors determining the carcinogenic potential of *pks^+^* bacteria.

## MATERIALS AND METHODS

### Cell lines, treatments, and synthetic colibactins

Colibactin 742 and 746 were prepared according to the method of Wernke et al. (12) and resuspended in DMSO. Lentiviral packaging was performed using the Lenti-X 293T Cell Line (Takara Bio, 632180). Genome-scale screening was performed using HEK293T cells (American Type Culture Collection [ATCC], CRL-3216). Validation was performed using HEK293T cells and HT-29 cells (ATCC, HTB-38). As a positive control in cell viability experiments, cell lines were treated with mitomycin C at a concentration of 3 µM. In all validation experiments, 0.01% DMSO was added as a negative control to replicate the volume of DMSO added during 10 µM colibactin 742 treatment. *cnf-1* gene presence in *Klebsiella pneumoniae* strain 51-5 was examined by PCR using published primers (PMID: 31139264; cnf-1 forward 5′-GGGGGAAGTACAGAAGAATTA-3′ and cnf-1 reverse 5′-TTGCCGTCCACTCTCACCAGT-3′). *E. coli* strain UM141 (42) was used as a positive control.

### RNA extraction, Illumina sequencing library construction, and sequencing

FHC cells were cultured in media containing HEPES (10 mM), insulin (0.005 mg/mL), epidermal growth factor (Peprotech, 20 ng/mL), and 10% fetal bovine serum (FBS). Cells were seeded at 2 × 10^5^ per well in six-well plates, treated with different concentrations of 742 or 746, and collected after 12 h exposure. For *Klebsiella pneumoniae* 51-5 infection, media were replaced with antibiotic free media, and cells were infected at an MOI of 50 for 4 h before being replaced with media supplemented with 200 µg/mL gentamicin for 18 h. Cells were collected by cell scraping in ice-cold phosphate-buffered saline (PBS), and total RNA was extracted using the RNeasy isolation kit with on-column Dnase treatment (Qiagen). RNA concentration was measured by the QUBIT fluorescent method (Invitrogen) and Agilent Bioanalyzer. Total RNA (250 ng) (RIN > 7) was used for library construction using the reagents provided in the NEBNext Poly(A) mRNA Magnetic Isolation Module (New England Biolabs) and the NEBNext Ultra II Directional RNA Library Prep Kit (New England Biolabs) according to the manufacturer’s user guide. Briefly, 250 ng of total RNA was used for mRNA enrichment using the NEBNext Poly(A) mRNA Magnetic Isolation Module (New England Biolabs). Then, the poly(A)-enriched RNA was fragmented in NEBNext First Strand Synthesis Buffer via incubation at 94°C. First-strand cDNA was synthesized using reverse transcriptase and a random hexamer primer followed by ds cDNA synthesis, end-repair and dA tailing. Illumina adaptors were ligated to the sample, amplified, and purified using AMPure beads (Beckman Coulter). The library size and mass were assessed using a High Sensitivity DNA1000 Screen Tape by Agilent TapeStation. Normalized libraries were treated with the Free Adapter Blocking Reagent (Illumina). The library pool was then diluted to 0.8 nM, and 120 pM was spiked with 1% PhiX. Thirty-nine barcoded libraries were pooled equimolarly for sequencing on a single 0.25 lane of Illumina NovaSeq 6000 S5 (2 × 150 cycles).

### RNA-seq analysis

Sequencing reads were quality checked and confirmed to be of high quality (sequencing quality score of 20 or more) then filtered to remove sequencing adaptors using Trimmomatic (v.0.39) (43). This resulted in an average of 51,357,114 reads per sample (25,678,557 reads for each end) that were then used for subsequent analysis. Alignment and gene expression quantification were done through STAR (v.2.7.9a) (44) using the human reference genome (GRCh38) with its corresponding annotation file obtained from the European Bioinformatics Institute (EMBL-EBI, http://ftp.ebi.ac.uk). More than 90% of the input reads for each sample were successfully aligned to GRCh38. Gene counts were then imported into edgeR (v.3.26, R v.4.1.2) (45) for normalization and differential expression analysis. We considered a transcript differentially expressed if its edgeR FDR adjusted *P* value was less than 0.05. Principal component analysis was done using the R prcomp function using a matrix of log-transformed values generated with the R rlog function with the option blind set to true. Heatmaps were generated using the R pheatmap package (https://CRAN.R-project.org/package=pheatmap). Pathway overrepresentation analysis was performed using the PANTHER classification system as previously described (46, 47). Significance was assessed using Fisher’s exact test with false discovery rate and mapped to the GO complete biological processes data set.

### Genome-wide CRISPR/Cas9 screening

Genome-wide CRISPR/Cas9 screening was carried out using the GeCKO v2 library targeting 19,050 human genes with over 123,411 individual guide RNAs (six sgRNAs per gene and 2,000 negative controls) as previously described (48). For the colibactin compound screen, due to limited availability of colibactin 742 and 746, a modified version of this protocol was used, in which a half library consisting of three sgRNAs per gene was used (GeCKO v2 library A). Lentiviral particles were prepared by transfecting HEK293FT cells (Thermo Fisher Scientific) with the sgRNA library, packaging (psPAX2, Addgene), and envelope (PSMD2.g, Addgene) plasmids, followed by harvesting media and concentrating the lentivirus using the Lenti-X Concentrator (Takara Bio). Lentiviral titer was confirmed using CellTiter-Glo (Promega). Cells were transduced at a lentiviral MOI of 0.3, and a total of 2.08 × 10^8^ HEK293T cells (full library) or 1.08 × 10^8^ (half library) HEK293T cells were transduced before 5 days of puromycin selection and expansion. Screens were carried out using a minimum of 325× genome coverage per condition, and all cell culture was carried out in DMEM supplemented with 10% FBS (D10). For the *K. pneumoniae* screen, cells were treated at an MOI of 50 for 4 h in antibiotic-free media before these cells were washed twice and the media was replaced with D10 supplemented with 200 μg/mL gentamicin. This process was repeated at each passage for a total treatment duration of 14 days and three infection cycles. For the colibactin compound screen, cells were grown in the presence of 5 μM colibactin 742 or 746 (inactive colibactin control) continuously for 12 days. Cells were passaged every 3 days with fresh colibactin 742 or 746 added at each passage, for a total duration of 12 days and four exposure cycles.

To amplify sgRNA tags, surviving cells were pelleted and DNA was extracted using the QuickDNA Midi-prep Plus kit (Zymo) before purification using a Zymo-Spin V column-based purification method as previously described (49). sgRNA tags were amplified using high-throughput PCR amplification using NEBNext High-Fidelity Taq Mastermix (New England Biolabs) and indexing primers NGS-Lib-KO-Rev 1-10 as previously described (48). Barcoded samples were pooled at equimolar concentrations and fragment size-selected at 260–270 bp. The library was sequenced using the NovaSeq 6000 platform (SP 2 × 100), and reads were analyzed using the MAGeCK robust rank aggregation algorithm to determine gene essentiality (49, 50).

### Single-target knockout generation

Oligos were inserted into the pLentiCRISPRv2 (Addgene) backbone using the Golden Gate Assembly protocol. The list of oligos used for single-target knockout validation are provided in Table 1. Briefly, the top and bottom oligos were annealed together at 100 μM each, ramping from 95℃ to 25℃ at 5℃/min. The annealed oligos were inserted into pLentiCRISPRv2 plasmid using the NEBridge Golden Gate Assembly Kit (BsmBI v.2) from New England Biolabs, according to the manufacturer’s instructions. Two microliters of the reaction mix was used to transform NEB Stable cells. Plasmids were prepped from individual colonies and sequenced with U6 forward primer to verify correct insertion of each oligo. Lentiviral particles were prepared, and HEK293T or HT-29 cells were transduced as previously described before being cultured for 5 days in the presence of 1.0 µg/mL puromycin. Knockout validation was carried out using Western blot or amplicon sequencing followed by TIDE analysis as previously described (19).

**TABLE 1:** Oligonucleotide sequences used for single-target CRISPR knockout in this study

Target gene	Identifier	GeCKOv2 UID	5′−3′ sequence
CRCP	81	HGLibA_11081	GTTACTGAGAAGCGCAGAAT
CRCP	82	HGLibA_11082	GACTGCTGTGGAGATCCAGC
PSMD4	171	HGLibA_39171	TAGGACTCACATGGGCCACG
PSMD4	172	HGLibA_39172	CTTCTGCACGGGCATCCGC

### Western blot

Cells were washed with cold PBS, collected by manual scraping, and pelleted by centrifugation at 400 × *g* at 4°C for 5 min. Cell pellets were resuspended in 100 μL complete radioimmunoprecipitation assay (RIPA) buffer with 1× cOmplete EDTA-Free Protease Inhibitor (Sigma-Aldrich) and incubated for 15 min on ice. Lysates were sonicated and incubated for another 15 min on ice and centrifuged for 5 min at 13,000 × *g* at 4°C before the resultant supernatant was used for Western blot. Proteins were separated by SDS-PAGE and transferred to polyvinylidene difluoride (PVDF) membranes (Bio-Rad). Membranes were incubated with primary α-tubulin (Cell Signaling Technology, 3873S; 1:2,000) or CRCP polyclonal antibody (Proteintech, 14348-1-AP; 1:500) antibodies. Secondary anti-rabbit or anti-mouse horseradish peroxidase-conjugated antibodies were visualized with chemiluminescent Pico (Thermo Fisher Scientific) enhanced chemiluminescent (ECL) substrate and imaged using a ChemiDoc XRS imager (Bio-Rad).

### Cell viability

Cells were seeded at a density of 8,000 cells per well in 96-well tissue culture plates overnight before 48 h treatment with the indicated colibactin compound. After 48 h, cell viability was measured using the CellTiter-Glo (Promega) luminescent cell viability assay using the manufacturer’s protocol using the CLARIOstar^Plus^ (BMG Labtech) microplate reader.

### γH2AX flow cytometry and cell cycle analysis

Cells were seeded at 200,000 cells per well in a six-well tissue culture plate overnight, after which they were incubated with colibactin 742 overnight or infected with *K. pneumoniae* at an MOI of 50 for 4 h in antibiotic-free media before the media were replaced with normal growth media supplemented with 200 μg/mL gentamicin overnight. Cells were collected in cold PBS, fixed, and permeabilized in 70% ethanol for a minimum of 2 h at −20°C. Cells were washed with bovine serum albumin (BSA)-T-PBS (PBS containing 1% BSA and 0.2% Triton X-100) and incubated with Alexa Fluor 647 anti-H2AX-phosphorylated (Ser139) antibody (BioLegend, diluted 1:200 in BSA-T-PBS) overnight at 4°C. Cells were washed with PBS and resuspended in 500 μL PBS for analysis. For cell cycle arrest, cells were fixed and permeabilized as described above before washing with BSA-T-PBS and resuspension in propidium iodide (Life Technologies) with 100 μg/mL RNase (Sigma-Aldrich). A minimum of 10,000 cells from each sample were analyzed using the Accuri C6 (BD Biosciences) flow cytometer, and data were processed and analyzed using FlowJo (BD Biosciences).

### Immunofluorescence detection of γH2AX

IEC-6 cells were seeded in eight-well chamber slides (Thermo Fisher Scientific) at 5 × 10^4^ cells per well and serum starved in 2% FBS medium overnight. Cells were treated with DMSO or the indicated concentration of 742 or 746 for 24 h before washing once with cold PBS and fixation in 4% methanol-free formaldehyde (Thermo Fisher Scientific) for 30 min on ice, washing with PBS, and quenching of free aldehydes with a 50 mM NH_4_Cl solution for 10 min. Cells were permeabilized using 0.25% Triton X-100 (Sigma-Aldrich) for 5 min on ice, washed with PBS, and blocked with blocking solution (PBS containing 1% BSA and 5% normal goat serum) for 1 h on ice and incubated with the phospho-histone H2AX (Ser139) rabbit monoclonal antibody (Cell Signaling Technology, 9718) diluted 1:800 in blocking solution overnight at 4°C. Cells were washed with PBS and incubated in the secondary Alexa Fluor 488 goat anti-rabbit IgG (H + L) antibody (Life Technologies, A-11034) for 45 min at room temperature. Cells were washed with PBS and mounted with VECTASHIELD mounting medium with 4′,6-diamidino-2-phenylindole (DAPI) (Vector Labs). Cells were examined for immunofluorescence using a Leica DM6000B upright microscope and counted as positive if more than five foci/nucleus were detected.

### Colony formation

NTC or *PSMD4*^−/−^ sg172 cell populations were treated with the indicated concentration of colibactin 742 for 24 h before dissociation and seeding. Soft-agar colony formation was performed as previously described (51). Briefly, a bottom layer of 1% noble agar (BD Biosciences) was overlaid with a 0.6% noble agar mixture containing 5 × 10^3^ cells/well in a six-well tissue culture plate, covered with cell culture media, and cultured at 37°C for 21 days. Colonies were stained with 0.1% crystal violet in 10% ethanol for 30 min before 5× rinsing with dH_2_O. Images were captured using the BioRad GelDoc XR+ with ImageLab software.

### SA-β-galactosidase staining

Two hundred thousand cells per well were seeded in a six-well plate with HT29 growth medium and incubated for 24 h, followed by treatment with DMSO or colibactin 742 (1 or 5 µM) for 24 h. Cytochemical staining for SA-β-galactosidase was performed using a Senescence β-Galactosidase Staining Kit (Cell Signaling Technology, #9860) at pH 6.0, with cells fixed using the provided fixative solution and incubated with the staining solution at 37°C overnight before brightfield imaging at ×20 magnification. All experiments were repeated three times.

### Cell proliferation 5-bromo-2′-deoxyuridine (BrdU) staining

Cell proliferation assay was performed in a 96-well plate by plating 50,000 cells per well and incubating at 37°C overnight in HT29 growth medium, followed by treatment with DMSO or colibactin 742 (1 or 5 µM) for another 24 h. Proliferation assays were carried out using the BrdU Cell Proliferation Chemiluminescent Assay Kit (Cell Signaling Technology, #5492). BrdU solution was added to each well and incubated for 24 h, followed by cell fixation, addition of the detection antibody, and measurement of chemiluminescence at 450 nm to assess cell proliferation.

### Statistical analysis

All statistical tests were performed using GraphPad Prism (v.6). All tests were two tailed; parametrical tests were used only for normally distributed data.

## Data Availability

Raw data from RNA-seq experiments are available in the National Center for Biotechnology Information (NCBI) Sequence Read Archive (SRA) with accession number PRJNA1074301. Raw data from CRISPR/Cas9 screening files are available in the NCBI SRA with accession number PRJNA1074360. All other data are available in the main text or the supplemental materials.

## SUPPLEMENTAL MATERIAL

Supplemental figures msphere.00692-24-s0001.docx Figures S1 to S5.

ASM does not own the copyrights to Supplemental Material that may be linked to, or accessed through, an article. The authors have granted ASM a non-exclusive, world-wide license to publish the Supplemental Material files. Please contact the corresponding author directly for reuse.
