# Supplementary material for: Genome-scale CRISPR/Cas9 screening reveals the role of PSMD4 in colibactin-mediated cell cycle arrest
Source: mSphere. 2025 Feb 7;10(3):e00692-24. doi: 10.1128/msphere.00692-24 (PMC11934320; doi:10.1128/msphere.00692-24)
Supplement: Supplemental figures — Figures S1 to S5. [file msphere.00692-24-s0001.docx]

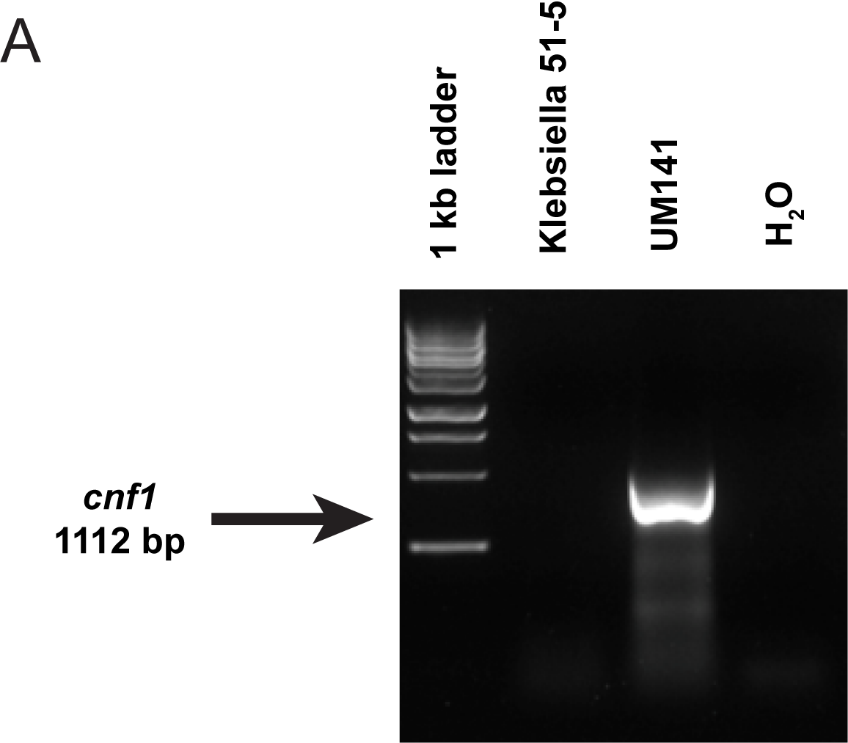


**Figure S1.** *Klebsiella pneumoniae* 51-5 does not produce cytotoxic necrotizing factor-1 (CNF1). A) PCR amplification targeting a 1112-bp amplicon of the *cnf1* gene in *K. pneumoniae* 51-5 (lane 2) and a *cnf1^+^ Escherichia coli* isolated from a human patient (UM141, lane 3).


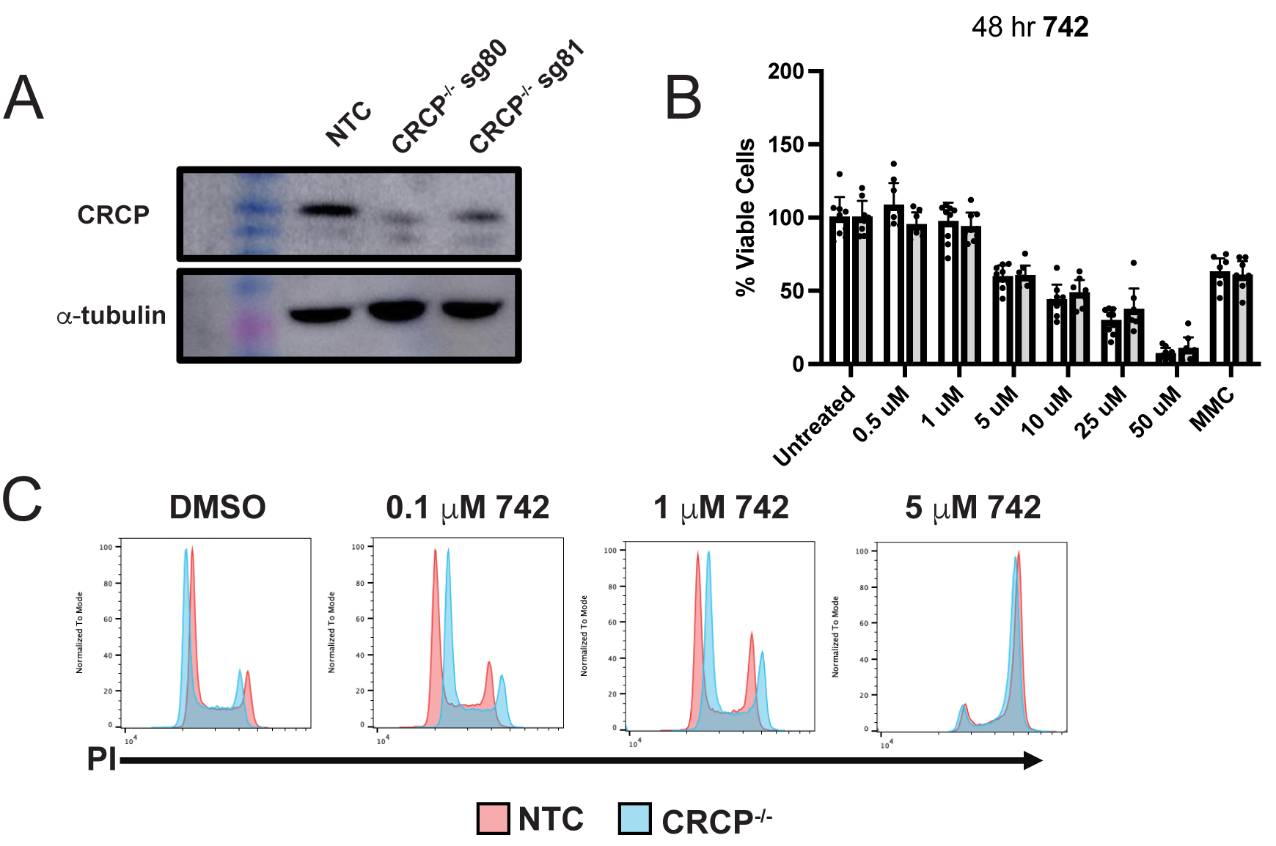


**Figure S2.** The calcitonin related gene-related peptide-receptor (*CRCP*) does not sensitize HEK293t cells to colibactin-induced cell cycle arrest. A) Validation of *CRCP* knockout in non-target control (NTC) and *CRCP*^-/-^ HEK293t cells after CRISPR/Cas9 gene editing. B) Cell viability in NTC and *CRCP*^-/-^ HEK293t cells after 48 hr. treatment with colibactin 742 at the indicated concentration. No statistically significant difference was observed after treatment with colibactin 742 at any concentration. C) Western blot of phosphorylated CDC2 in NTC and *CRCP*^-/-^ HEK293t cells treated with DMSO, MMC, or colibactin 742 at the indicated concentration for 24 hours. D) Flow cytometry histograms of propidium iodide (PI) staining in NTC or *CRCP*^-/-^ HEK293T cells after 24 hr DMSO or colibactin 742 treatment.


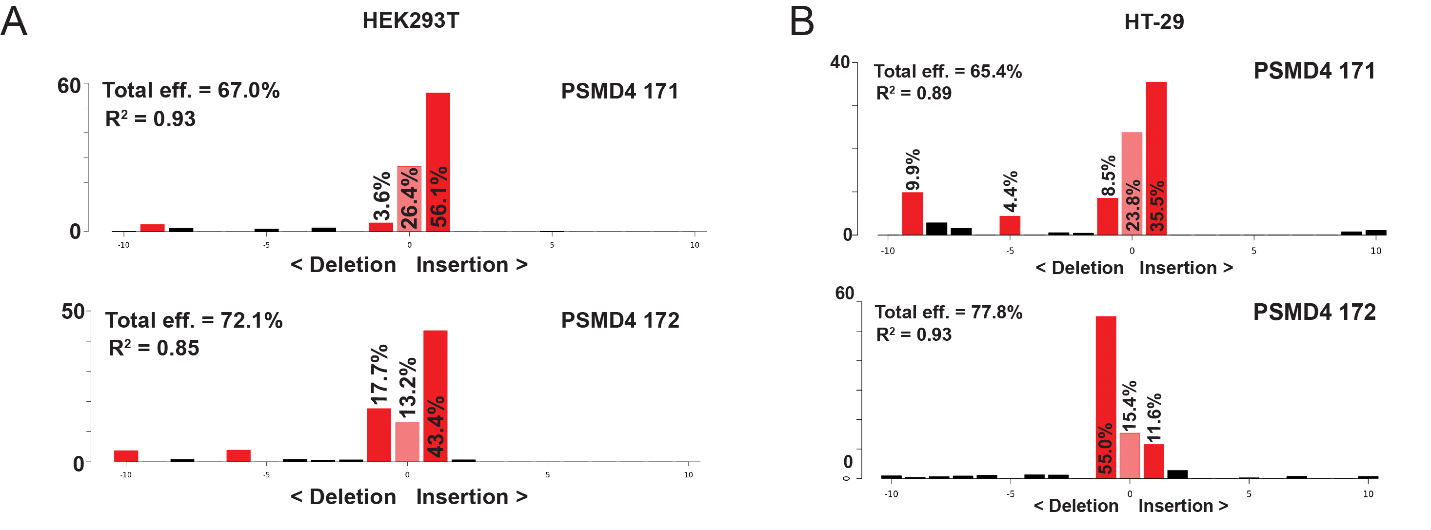
**Figure S3.** Validation of *PSMD4* knockout in HEK293t and HT-29 cells after CRISPR/Cas9 gene editing. A) Indel spectrum quantified by TIDE analysis of HEK293t populations after CRISPR/Cas9 with two sgRNA sequences from the GeCKOv2.0 library targeting *PSMD4*. Total knockout efficiency calculated as *R^2^* x 100% wild type. B) Indel spectrum quantified by TIDE analysis of HT-29 populations after CRISPR/Cas9 with two sgRNA sequences from the GeCKOv2.0 library targeting *PSMD4*. Total knockout efficiency calculated as *R^2^* x 100% wild type.


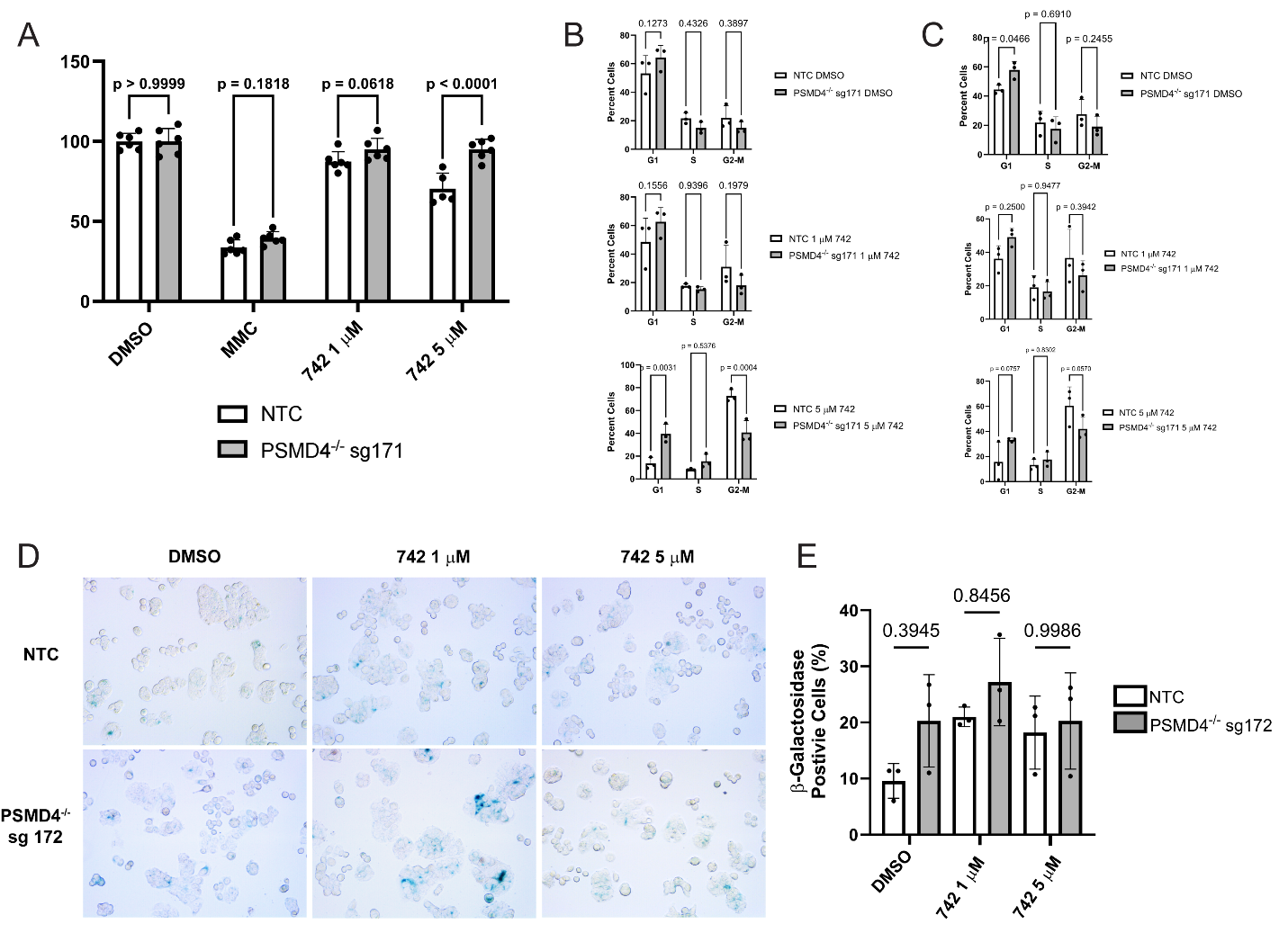
**Figure S4.** Validation of reductions in cell viability and G2-M cell cycle arrest using a second sgRNA targeting *PSMD4.* A) Cell viability in after 48 hr. treatment with DMSO, mitomycin C, or colibactin 742 at the indicated concentration, in non-target control or *PSMD4*^-/-^ HEK293t cell populations. n=6 from a single experiment, 2-way ANOVA with Tukey’s post-hoc test.

facilitates colibactin-induced G2-M cell cycle arrest. B) Percentage of NTC or *PSMD4*^-/-^ HEK293T cells in G1, S, and G2-M phase after treatment with 24 hr DMSO, or colibactin 742. n=3 independent experiments, 2-way ANOVA with Tukey’s post-hoc test. C) Percentage of NTC or *PSMD4*^-/-^ HT-29 cells in G1, S, and G2-M phase after treatment with DMSO, or colibactin 742. n=3 independent experiments, 2-way ANOVA with Tukey’s post-hoc test. D-E) Representative images (D) and quantification of β-galactosidase positive cells (E) in NTC and *PSMD4*^-/-^ HT-29 cells after overnight treatment with DMSO or colibactin 742. n=3 independent experiments, 1-way ANOVA with Tukey’s post-hoc test, all comparisons with p<0.1 are shown.


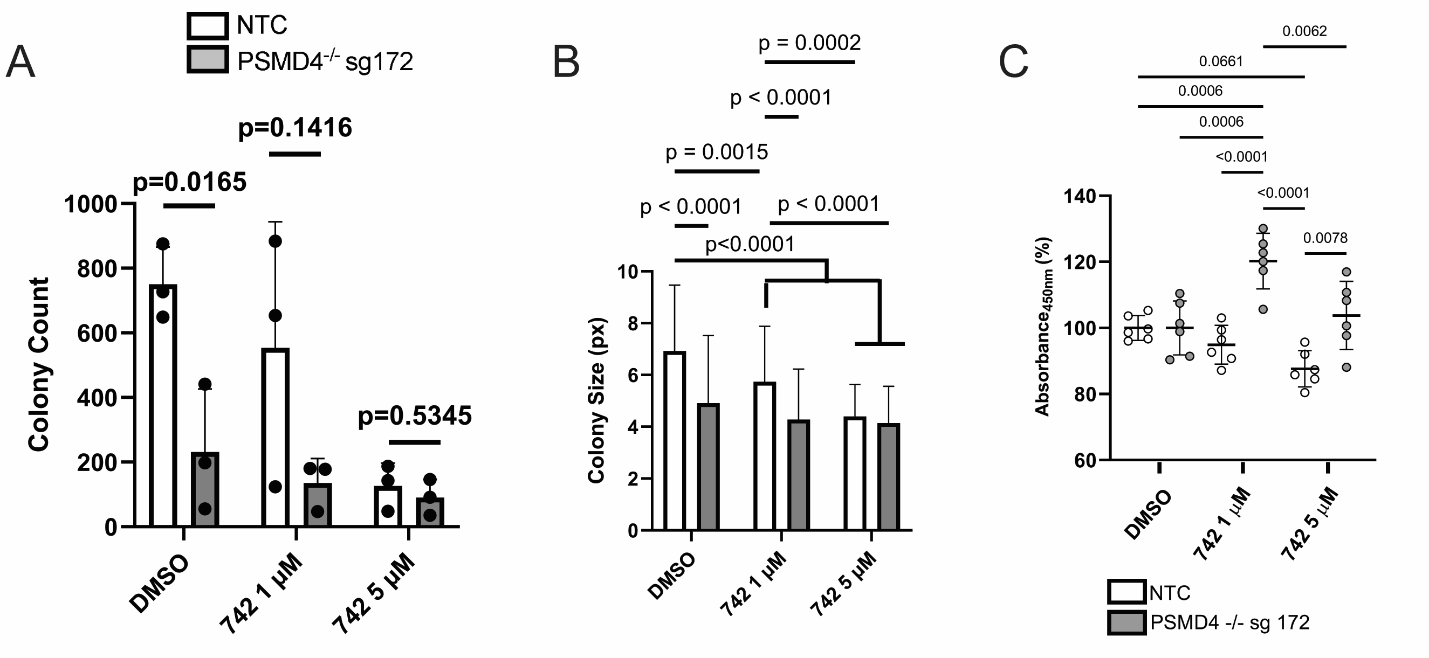
**Figure S5.** *PSMD4* facilitates colony formation but sensitizes cells to colibactin-induced reduction in colony forming ability. A) Quantification of colony formation in non-target control (NTC) and *PSMD4*^-/-^ HT-29 cells soft agar 21 days after 24 hr DMSO or colibactin 742 treatment, with each data point representing the number of colonies in a single 9.6 cm^2^ well. n=3 independent experiments, unpaired *t*-test. B) Quantification of colony diameter in NTC and *PSMD4*^-/-^ HT-29 cells soft agar 21 days after 24 hr DMSO or colibactin 742 treatment, n=90 randomly selected colonies pooled from three independent experiments (30 colonies per experiment), 2-way ANOVA with Tukey’s post-hoc test. All comparisons with p<0.05 are shown. C) Quantification of the relative proportion of BrdU-positive cells in NTC and *PSMD4*^-/-^ HT-29 cells after overnight treatment with DMSO or colibactin 742, n=6 results from a single experiment, representative of two independent experiments. One-way ANOVA with Tukey’s post-hoc test.
